# A novel testate amoebae trait-based approach to infer environmental disturbance in *Sphagnum* peatlands

**DOI:** 10.1038/srep33907

**Published:** 2016-09-23

**Authors:** Katarzyna Marcisz, Daniele Colombaroli, Vincent E. J. Jassey, Willy Tinner, Piotr Kołaczek, Mariusz Gałka, Monika Karpińska-Kołaczek, Michał Słowiński, Mariusz Lamentowicz

**Affiliations:** 1 Laboratory of Wetland Ecology and Monitoring, Adam Mickiewicz University, Krygowskiego 10, 61-680 Poznań, Poland; 2 Department of Biogeography and Palaeoecology, Adam Mickiewicz University, Krygowskiego 10, 61-680 Poznań, Poland; 3 Institute of Plant Sciences and Oeschger Centre for Climate Change Research, University of Bern, Altenbergrain 21, CH-3013 Bern, Switzerland; 4 Ecole Polytechnique Federale de Lausanne, School of Architecture, Civil and Environmental Engineering, Ecological Systems Laboratory, Lausanne CH-1015, Switzerland; 5 WSL – Swiss Federal Institute for Forest, Snow and Landscape Research, Site Lausanne, Station 2, Lausanne CH-1015, Switzerland; 6 Department of Environmental Resources and Geohazards, Institute of Geography and Spatial Organisation, Polish Academy of Sciences, Twarda 51/55, 00-818 Warszawa, Poland

## Abstract

Species’ functional traits are closely related to ecosystem processes through evolutionary adaptation, and are thus directly connected to environmental changes. Species’ traits are not commonly used in palaeoecology, even though they offer powerful advantages in understanding the impact of environmental disturbances in a mechanistic way over time. Here we show that functional traits of testate amoebae (TA), a common group of palaeoecological indicators, can serve as an early warning signal of ecosystem disturbance and help determine thresholds of ecosystem resilience to disturbances in peatlands. We analysed TA traits from two *Sphagnum*-dominated mires, which had experienced different kinds of disturbances in the past 2000 years – fire and peat extraction, respectively. We tested the effect of disturbances on the linkages between TA community structure, functional trait composition and functional diversity using structural equation modelling. We found that traits such as mixotrophy and small hidden apertures (plagiostomic apertures) are strongly connected with disturbance, suggesting that these two traits can be used as palaeoecological proxies of peatland disturbance. We show that TA functional traits may serve as a good proxy of past environmental changes, and further analysis of trait-ecosystem relationships could make them valuable indicators of the contemporary ecosystem state.

Understanding the response of ecosystems to climate and human driven processes under recent global change conditions is an important challenge for scientists^1^,^2^. Patterns of mean annual temperature and precipitation will change^3^ and climatic extremes may cause environmental stress and disturbance which may lead to shifts in ecosystem structure and function^4^,^5^,^6^. Therefore, it is vital to better understand ecosystem vulnerability and resilience to disturbances, and a long-term view of ecosystem development and dynamics using biotic proxies is especially beneficial^7^,^8^. Among endangered ecosystems, peatlands are important carbon (C) sinks^9^. Water deficit negatively influences their C storage capacity by modifying the hydrophysical properties of peat^10^, which affects peat-forming processes, potentially leading to increased C emission to the atmosphere^11^,^12^.

Anthropogenic activities, such as peat extraction, drainage and burning, are among the most common water disturbances in peatlands leading to substantial shifts in *Sphagnum* and vascular vegetation cover^13^,^14^, which then feedback into C fluxes^15^. Moreover, intensive drainage promotes prolonged anthropogenically-induced drying and significantly increases peatland vulnerability to fire^16^. Burning is considered to be a major force affecting various ecosystems^17^, and is especially important for peatlands, in which fires often cause long-lasting smouldering combustion that results in fuel consumption and substantial CO_2_ emission^9^,^18^. It has been lately shown that even moderate drops in the water table can lead to increased vulnerability of peatlands to wildfire^16^. As fire activity has significantly increased in recent years in many parts of the world^19^, studying the vulnerability of peatlands to fire disturbance over long time scales may provide new insights for assessing future peatland responses under global-change scenarios.

Among the different approaches used in palaeoecology, a functional trait-based approach is a useful tool to track ecosystem response to climatic and anthropogenic change^20^,^21^. Species’ functional traits have been shown to be closely related to ecosystem processes and functioning through evolutionary adaptation^22^,^23^, and they closely reflect species interactions in the communities and their response to changing environmental conditions^20^. Together with the analysis of functional traits, functional diversity is substantial as it represents the diversity of species functions, and is thought to be a better proxy for ecosystem functioning than the number of species *per se*^21^,^24^ or taxonomical diversity, which refers to the number and the relative abundance of species in the community. Additionally to conventional taxonomical analyses, species’ functional traits and functional diversity may serve as an early signal of ecosystem disturbance, helping determine thresholds of ecosystem resilience to disturbances. Such information might be crucial for nature conservation and biodiversity protection^21^; however, an important challenge is to choose which traits from which fossil record can be used in both palaeoecology and modern ecology to explicitly link the past with the present and unambiguously infer peatland response to environmental changes^25^. In the peatland microbial food web, testate amoebae (TA) are crucial for ecosystem functioning^26^, and they are key players in the elements cycling in soil^27^. TA are useful indicators of hydrological changes in peatlands^28^, as due to preservation of their tests it is possible to quantitatively reconstruct past water table depth^29^,^30^. TA functional traits have recently been used in ecological and palaeoecological studies of peatlands and have been linked to specific ecosystem functions: in ecological studies strong relationships have been found between moss type and TA size-structure^31^, and the warming effect is positively correlated to shell-aperture size over body size ratio^32^. Palaeoecological studies have underlined the crucial role of the shell aperture position for TA adaptation to hydrological conditions^33^, showed that drying eliminated large TA species from the community^34^, and that past atmospheric pollution and dust deposition influenced shell size and structure^35^.

Still, there is a scarcity of studies focusing on the long-term impact of disturbances on testate amoeba trait composition, in particular concerning effects of human-induced disturbances. Therefore, our primary goal is to examine whether the response of TA to various environmental disturbances over the past 2000 years is reflected in their trait composition. We aim to assess differences between TA trait groups in response to two disturbance agents: fire and peat extraction. We also want to define the relationship between the taxonomical and functional diversity for TA. We hypothesised that TA community composition responds to abrupt disturbance. In particular, we expected that certain traits, such as species size or aperture position, reflect shifts in peatland hydrology, based on recent findings studying the effect of water level changes^33^. Finally, we tested the effect of disturbances on the linkages among the structure of TA communities, the composition of functional traits and functional diversity. With this study we want to provide information about the response of TA traits to disturbances, which could support conventional microscopy analysis of TA communities.

## Methods

### Study sites and palaeoecological datasets

To investigate the long-term response of testate amoeba communities to disturbances, we used existing palaeoecological data sets from two *Sphagnum* peatlands located in Poland: Linje^13^ and Puścizna Krauszowska (PK)^36^,^37^. Linje, located in northern Poland (90 m a.s.l., 53°11′15″N, 18°18′34″E), is classified as a poor fen^38^, covering an area of 5.95 ha^39^. PK, located in the Orawa-Nowy Targ Basin, Polish Western Carpathians (613 m a.s.l., 49°28′06″N, 19°56′18″E)^36^, is a bog covering an area of 79 ha. Both peatlands were significantly disturbed by humans and those disturbances resulted in peatland drying. Environmental changes in the Linje mire were linked with the establishment of human settlement and a parallel increase in fire activity that led to substantial vegetation change in the mire area in the 14^th^ century^13^. In PK, anthropogenically-induced drying appeared due to drainage followed by peat extraction that led to peat layer disruption from the 7^th^ to the beginning of 19^th^ century AD (this layer does not have an absolute chronology due to radiocarbon date inversion)^36^,^37^. Two hundred years ago, drainage ditches have been established on both mires, and a growth of local population was recorded in the last 100 years^38^,^40^. Such disturbances allow us to cover a large gradient which can better support our research questions and test our hypotheses.

Selected peat cores cover the last 2000 years, and possess TA community data and associated depth to water table (DWT) reconstructions, and macroscopic charcoal, plant macrofossil and pollen data^13^,^36^,^37^ (Supplementary Methods S1). The chronology relies on depth-age models with 20 radiocarbon dates on terrestrial plant macrofossils and 56 lead-210 dates for Linje^13^, and 12 radiocarbon dates on terrestrial plant macrofossils and 18 lead-210 dates for PK^37^ (Supplementary Table S2, Supplementary Figure S3).

### Selection of testate amoeba functional traits

In order to understand the response of TA communities to the past disturbances on the group level and community level, we exploited: (1) *diversity indices of the testate amoeba functional groups within the community*; and (2) *functional traits of the testate amoeba community*.

**Figure 1:**
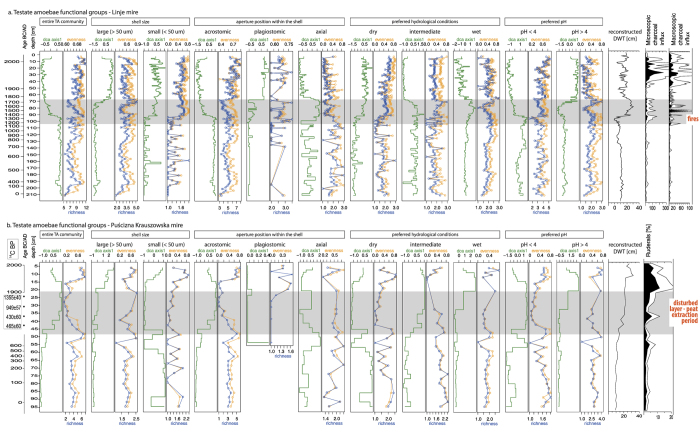
Values of diversity indices (evenness, richness, compositional change-DCA axis 1) of the testate amoeba functional groups within the testate amoeba communities calculated for Linje (**a**) and Puścizna Krauszowska (**b**) mires, associated depth to water table reconstructions (cm) and disturbance indicators: charcoal influx (particles/cm^2^/year, proxy for fire) and pollen indicators linked to human activities, i.e. released by ruderals (%, proxy for direct human impact, including *Ambrosia artemisiifolia* type, *Artemisia*, Brassicaceae, Chenopodiacae, *Medicago lupulina* type, *Plantago lanceolata*, *P. major*, *P. media*, *Polygonum aviculare* type, *Rumex acetosa* type, *R. acetosella* type, *R*. cf. *obtusifolius*, *Urtica*^62^). Disturbance periods are marked in grey.

**Figure 2:**
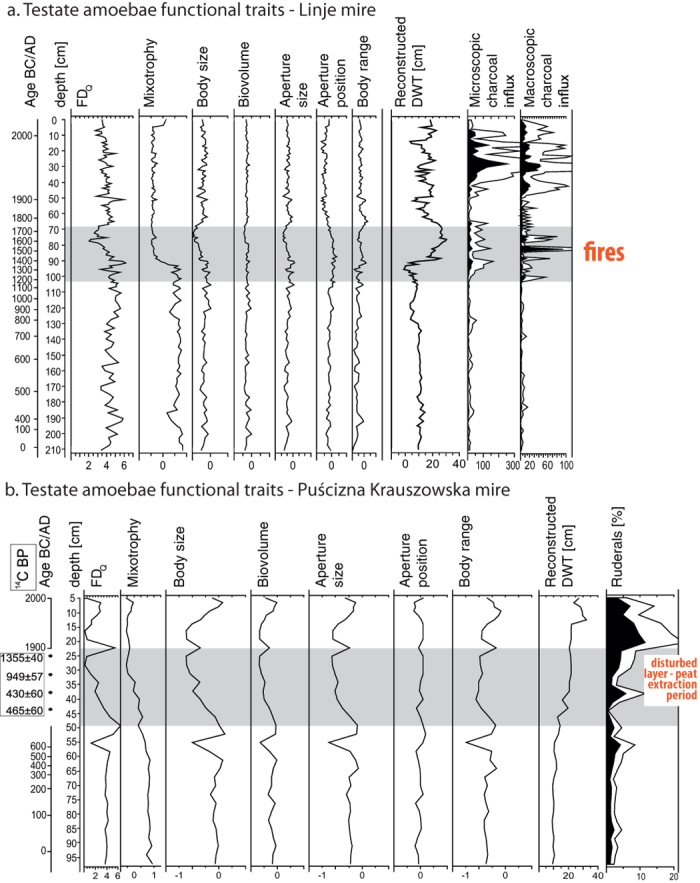
Results of functional traits and functional diversity (FD_Q_) of the testate amoeba communities calculated for Linje (**a**) and Puścizna Krauszowska (**b**) mires, associated depth to water table reconstructions (cm) and disturbance indicators: charcoal influx (particles/cm^2^/year, proxy for fire) and ruderals (%, proxy for direct human impact). Disturbance periods are marked in grey.

1. Values of *diversity indices for testate amoeba functional groups* (evenness, richness and compositional change-DCA axis 1) along the sequences were calculated for selected TA groups (Fig. 1). Comparing evenness and richness is useful to reconstruct past trends in diversity and to assess how the diversity is determined by specific groups of taxa or individual species^41^. This approach was used to define if tendencies in certain groups can explain general changes in TA community, and to assess if particular groups of TA responses are faster than community change. TA groups were selected according to the ecological preferences of each species: preferred hydrological conditions and pH, and to the shell morphology: shell size and position of the shell aperture (acrostomic aperture – terminal, located on top of the shell, plagiostomic aperture – sub-terminal, hidden in the side of the shell, and axial aperture – located in the centre of the shell) (Supplementary Table S4).
2. *Functional traits of the testate amoeba community* were calculated for both profiles. Six traits were chosen for this study following Lamentowicz *et al*.^33^: mixotrophy, body size, biovolume, diameter of the shell aperture, position of the shell aperture within the shell and body range (Fig. 2). Descriptions of selected traits, their functions and predicted effects of disturbances on traits are listed in Table 1.

### Numerical analyses

Based on the TA functional traits, we calculated the community weighted mean values (CWM) of each standardised trait, which is an index of functional composition expressed as the mean trait value of species present in the community, weighted by their relative abundances^42^. Rao quadratic entropy (Q) was used as a measure for functional diversity (FD_Q_ value, Fig. 2)^43^,^44^. FD_Q_ is unit-less and shows how distinct communities are functionally different, i.e. increasing Rao’s Q values indicate functionally increasingly different communities. FD_Q_ was calculated using the synthetic functional traits (i.e. species coordinates on Principal Coordinate Analysis (PCoA) axis) defined above and the relative abundance of species^44^. To calculate synthetic functional traits, we created a functional distance matrix by applying Gower’s distance on each pair of species described by their traits, and then computed a PCoA on this basis^45^. Then, the first three axes of the PCoA (more than 80% of variance) were selected as synthetic functional traits summarising TA functional space, and species coordinates in the three-dimensional space defined by the PCoA were used to calculate FD_Q_.

**Figure 3:**
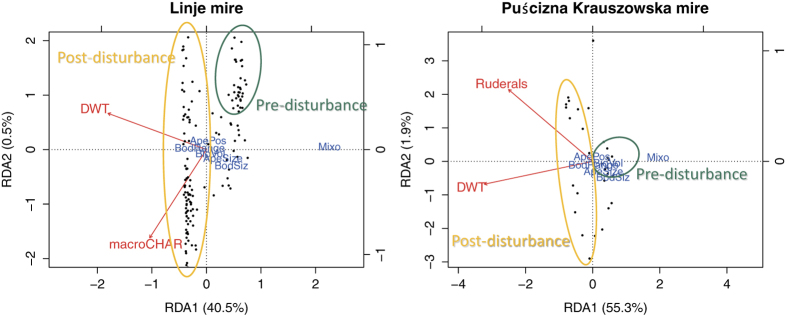
Redundancy analysis (RDA) showing the relationships between testate amoeba functional traits and environmental variables: macroscopic charcoal influx (proxy for local fire), ruderals (proxy for direct human impact) and reconstructed depth to water table (DWT), constructed for both data sets: Linje and Puścizna Krauszowska mires. The models are significant (*P *= 0.001 for each model, see Table 2). Black points represent individual samples. Green circles indicate samples from pre-disturbance communities; yellow circles indicate samples from post-disturbance communities. Abbreviations used in the figure: Mixo – mixotrophs; BodSiz – body size; BioVol – biovolume; ApeSize – diameter of the shell aperture; ApePos – shell aperture position; BodRange – body range; DWT – reconstructed depth to water table; macroCHAR – macroscopic charcoal influx/accumulation rate; ruderals – pollen human indicators.

**Figure 4:**
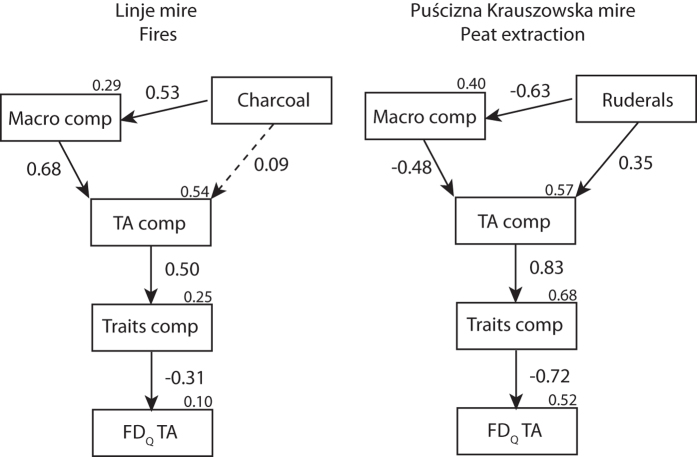
Structural equation models showing the effect of environmental disturbances on testate amoeba functional diversity (FD_Q_). Solid lines are significant paths (*P *< 0.05), while dashed lines represent non-significant paths (*P *> 0.05). Squared multiple correlations (*R*^*2*^) for the predicted/dependent factors are given on the top of the box of the dependent variable. TA comp = testate amoebae species composition (PCA axis 1); Macro comp = plant macro-remains composition (PCA axis 1); Traits comp = testate amoebae trait composition (PCoA axis 1). These threes variables are synthetic variables (see methods). Models include macroscopic charcoal influx (proxy for local fire) and ruderals (proxy for direct human impact) used as disturbance indicators for fire and direct human impact, respectively.

To see if functional diversity of TA communities responds similarly to disturbances in both datasets, redundancy analysis (RDA) was performed using six functional traits of the TA community and DWT, macroscopic charcoal influx and pollen indicators linked to human activities, i.e. released by ruderal plants as explanatory variables (Fig. 3, Table 2). We later used structural equation modelling (SEM)^46^ to test the effect of different disturbances on the linkages among TA community structure, functional trait composition and functional diversity, and on local vegetation composition (plant macro-remains) (Fig. 4). Using *a priori* knowledge, we developed several hypothetical mechanisms to build a network of causal relationships including predicted effects of disturbances^46^ (Table 1). We hypothesised within this causal network that environmental disturbances influence the TA community structure both directly and indirectly through its effect on local vegetation composition. We assumed that TA community structure drives its trait composition, and thus its functional diversity (FD_Q_). We used pollen human indicators as a proxy for peat extraction and macroscopic charcoal influx as a proxy for fire. For each core, we ran a DCA on a TA data set and extracted DCA site scores as a proxy for TA community composition. Similarly, we ran a PCA on CWM traits of TA of each core and extracted PCA site scores as a proxy for trait community composition. This model was tested for fire (n = 146) and human (n = 35) disturbances, respectively. Adequate model fits are indicated by non-significant χ^2^ tests (*P* > 0.05), a low Standardised Root Mean Square Residual index (SRMR < 0.1), and a low Root Mean Square Error of Approximation index (RMSEA < 0.1). Detailed pollen data used for both profiles can be found in Kołaczek, *et al*.^36^ and Marcisz, *et al*.^13^. All numerical analyses were conducted with R 3.0.1^47^ using the packages *FD*^42^, *sem*^48^ and *vegan*^49^.

## Results

### Diversity indices of the testate amoeba functional groups

The compositional change of TA showed a drastic turnover in both peatlands in response to disturbances; however, this occurred with a delay (Fig. 1). In Linje, the decrease in compositional change started in cal. AD 1390, after local fires appeared on the mire and the values decreased steadily until cal. AD 1700. Disturbed layer in PK also revealed decrease in TA community richness. It should be mentioned that communities in the disturbed layer might have partly been a result of secondary colonisation by TA (mainly dry indicators) after the peat disturbance. Post-disturbance TA communities stabilised in Linje. TA community composition in both peatlands remained quite stable and no community resilience can be observed. Evenness and richness showed parallel trends. In Linje they rose to cal. AD 400, when a short dry shift was recorded. The lowest values were recorded at cal. AD 620, and these rose again along with water table increases. During the fires, evenness and richness reached the highest values at cal. AD 1390, and then decreased. After the period of increased fire activity (from cal. AD 1700), the values were slightly variable, with a general rising trend. A similar situation was observed in PK, where evenness and richness were consistent along the core reflecting minima in disturbed layer. Post-disturbance (since cal. AD 1900) evenness and richness values were low; they started rising when the peatland was regenerating from disturbances (cal. AD 1965).

Compared to the community response, the response of some groups was delayed. The change of shell size in response to disturbance is clearly visible in Linje, where at the beginning of local fire activity richness and evenness of small species increased (cal. AD 1390). Simultaneously, evenness and richness of large TA slightly declined. In general, richness and evenness of large TA followed the community trend, whereas in the group of small TA evenness and richness varied before the fire, and after the fire they show similar tendencies. In PK, evenness and richness of large TA followed the community trend up to the disturbed layer; together with disturbance and post-disturbance evenness and richness of small TA was in line with the community response.

Species with acrostomic apertures were the most abundant in both data sets. In Linje, evenness and richness were very similar to community trends; during fires values decreased, and increased post-disturbance. Similarly, in PK richness and evenness followed community trends. In both peatlands, TA possessing axial apertures were highly variable. In pre-disturbance communities, TA with plagiostomic aperture were either not present in the community (PK), or their abundances were low (Linje).

In Linje, evenness and richness of TA preferring dry and intermediate water tables were variable pre-disturbance (up to cal. AD 1390), but during local fires indices values were more stable and began to change post fire. Evenness and richness of wet indicators were more stable post-disturbance, having the biggest variations during and after disturbances. In PK, the highest pre-disturbance (cal. AD 0–150) richness and evenness were observed for TA preferring intermediate and low water tables. Evenness and richness of all groups (especially dry TA) decrease in the layer disturbed by peat extraction.

Compositional change and changes in evenness and richness of TA preferring lower pH were consistent with the general community trends in both peatlands. Species preferring higher pH were less abundant and in PK compositional change was observed in the post-disturbance period. In Linje, the group of TA preferring high pH shifted during disturbances and stabilised post-disturbances.

### Functional traits of the testate amoebae and community patterns

The analysis of TA functional traits revealed similarities in community response to human induced disturbances: fire and peat extraction. In both datasets, FD_Q_ values decreased after disturbances started (stronger decreases are noted for PK), indicating that TA communities were functionally more similar during disturbances. In general, this shift was mainly caused by a decline in mixotrophs and body size (Fig. 2). Both peatlands were in stable hydrological conditions in the past (until cal. AD 1390 in Linje and cal. AD 650 in PK) and during that time TA traits were only slightly variable. In Linje mire, a slight decrease in water table in cal. AD 400 was simultaneous with a drop in mixotrophs and body range increase. At the beginning of fire activity in Linje mire (cal. AD 1225) and at the bottom of the disturbed layer in PK, trait values changed. In particular, we found a sharp decrease in mixotrophs in both mires, although this was more pronounced in Linje. Simultaneously, shell size and aperture size dropped. However, the biovolume remained quite stable. In PK, the number of mixotrophs decreased together with body size, aperture size and biovolume at the bottom of the disturbed layer. The response of TA communities in PK was not as rapid as in Linje; however, it is not possible to quantify this precisely, as the disturbed layers possess a disturbed chronology and they were not taken into account for depth-age modelling^37^. In both peatlands in the disturbed layer, large taxa and mixotrophs were eliminated from the community, while the number of small taxa increased. Post-disturbances in both peatlands, body size and aperture size increased, together with a slight increase in biovolume.

We used RDA ordination to explore the relationships between TA traits, DWT, ruderals and macroscopic charcoal influx, to define possible similarities in TA trait composition in peatlands that have been under different stressors. RDA models are highly significant (Fig. 3, Table 2), and they show comparable patterns of TA trait composition in both profiles, connected to hydrological (dry-to-wet) gradient. Mixotrophs are connected to pre-disturbance communities and high water table (Linje and PK), and negatively connected to macroscopic charcoal (Linje).

SEMs revealed that functional diversity of TA was significantly influenced by both fire and human disturbance (peat extraction) (Fig. 4). The effect of disturbance factors on TA is, however, different for both datasets. Fire disturbance explained only 10% (*R*^*2*^ = 0.1) of TA functional diversity, while human disturbances explained 52% (*R*^*2*^ = 0.52) of TA functional diversity (Fig. 4). In Linje, SEMs indicate that fire indirectly influenced TA functional diversity through its effect on vegetation composition (path = 0.53), which was then positively linked to TA community composition (0.68). TA community composition then significantly influenced the CWM trait composition (0.50) and functional diversity of TA (−0.31). In PK, human disturbances negatively correlated to macrofossil composition (−0.63) and positively to TA composition (0.35). The model also showed that plant macrofossil composition was a driver of TA composition (−0.48). Shifts in TA composition then strongly drove TA trait composition (0.83) and functional diversity (−0.72).

## Discussion

The analysis of TA functional traits revealed similar tendencies in both peatlands (Fig. 2), comparable with changes observed in the compositional change, evenness and richness indices along the studied datasets (Fig. 1). We found the largest variations of traits and indices together with or after human disturbance. Compositional change of TA community was recorded in both peatlands when disturbance started and TA communities were functionally more similar under the influence of stressors (Figs 1 and 2). Post-disturbances, TA communities remained stabilised. Compared to community patterns, we observed a delay in the response of some functional groups of TA (e.g. large TA or TA preferring pH > 4 in Linje, in case of which compositional change was observed when fires terminated, Fig. 1). However, in disturbed layers, the drop in the water table is simultaneous with a decrease in body size and aperture size, with no significant variations in biovolume (Fig. 2).

Analysis of TA functional groups revealed that richness and abundances of small TA and species preferring dry conditions were higher just after the influence of local fire, but decreases in the abundance of large TA and those preferring wet conditions were recorded with a short delay. This delay in response of wet indicators was also recorded in the cross-correlation analysis, i.e. of macroscopic charcoal influx with wet indicator species *Heleopera petricola*^13^, and led to the coexistence of wet and dry communities at the beginning of the local fire activity period. However, a clear coexistence of wet and dry communities was not recorded in PK, where the most abundant taxa were TA preferring intermediate water tables. This suggests lower water table seasonal variability and gradual water table lowering in the disturbed layer or, possibly, a combination of pre-disturbance communities established in this layer and those that colonised after the disturbance. In wet conditions, the co-occurrence of small and large TA is possible because they occupy different trophic niches with different functions in the microbial food web. Large TA are usually top predators feeding mostly on rotifers, nematodes, ciliates or other (smaller) TA, whereas small TA feed on small organisms that can be consumed through small apertures, mostly algae, fungi and bacteria^32^,^50^. In PK, high numbers of small taxa were recorded in the peat layers, where increased frequencies of fungi were recorded (personal communication with M. Karpińska-Kołaczek, May 2016). The coexistence of small and large TA taxa was already recorded in observational and experimental studies, and this was linked to peatland micro-topography^34^,^39^,^51^ and thus different habitats. TA preferring dry conditions mostly live on elevated hummocks, whereas TA preferring wet conditions live in pools. Fire on the peatland surface results in the removal of dry hummocks, whereas fire in the surrounding forest leads to deforestation, runoff changes and temporal peatland surface flooding^52^, as well as to increased dust deposition on the peatland surface^35^. Peatland flooding after fire is suitable for TA preferring wet conditions, but TA preferring dry conditions can still survive temporal increases in wetness. After the runoff changes and water loss is observed on the surface of the mire, microhabitats suitable for TA preferring wet conditions rapidly shrink, whereas those appropriate for TA preferring dry conditions are enlarged.

During the post-disturbance period in PK, large TA reappeared in low abundances and their evenness and richness decreased. In Linje, however, large TA got richer in the final stage of disturbances, but when fire activity decreased their evenness and richness decreased again. When disturbances were terminated, larger TA were not as abundant as before the disturbances and failed to dominate the community. This may be due to mire size, as small mires (e.g. Linje) possess low levels of variety and numbers of microhabitats. Therefore, when TA species become extinct, individuals from other habitats cannot colonise quickly. In contrast, in larger mires with variable microhabitats present and bigger and more diverse species pool for potential immigrants, such as PK, the possibility for faster colonisation from other microhabitats present on the peatland surface is higher. Another important factor is species size. Large species are heavier than smaller ones^53^,^54^; therefore, it is unlikely that they will colonise suitable habitats quickly. Wanner and Xylander^55^ observed that small Euglyphid TA were present in all succession stages, whereas large taxa occurred in later succession stages. In *Sphagnum* peatlands the most common Euglyphids are *Euglypha* sp., *Assulina* sp., *Trinema* sp. and *Corythion* sp.^56^. Tests of *Euglypha* sp. are built of easily dissolvable silica plates^56^; therefore, in palaeoecological reconstructions they are most often found only in the recent top peat layers^52^. Small *Trinema* sp. and *Corythion* sp. taxa were present in Linje and PK TA communities during and after disturbances, which suggests that small species do indeed colonise available niches rapidly and that they are not discouraged by tough environmental conditions.

Structural equation modelling provides a synthesis of the relationships between different proxies^33^. Using SEM, Lamentowicz *et al*.^33^ revealed that TA aperture position closely reflects water table variations. Similarly, we show that TA trait composition is in general affected by disturbances; however, this was more evident when peat extraction appeared (Fig. 4). In the case of fire, the composition of plant macrofossils on the mire surface was influenced first – a decrease in the abundance of *Sphagnum* and increase in abundance of *Eriophorum vaginatum* was recorded, and later TA were affected (see Figs 3 and 4 in Marcisz *et al*.^13^). Exploration of TA traits enabled us to confirm the previous findings of Fournier *et al*.^34^, who recorded that drying caused a decrease in species size and biovolume, and of Laggoun-Défarge *et al*.^53^, who found that larger species were more abundant in unexploited (undisturbed) sites on peatland than in cut-over parts.

Moreover, our findings show that mixotrophy and plagiostomic aperture traits were connected with local disturbances and, hence, we suggest that these traits could be considered disturbance indicators in palaeoecology. Mixotrophy was connected with pre-disturbance communities, where in periods of high water tables high numbers of mixotrophs (mainly *Hyalosphenia papilio*, *Archerella flavum*, *Heleopera sphagni* and *Amphitrema wrightianum*) were recorded in both peatlands (Fig. 3). Mixotrophs were lost during disturbances and they dropped rapidly in Linje. This underlines how sensitive mixotrophs are to environmental stressors including anthropogenically-induced drying^39^, and this is in accordance with previous research that proved their sensitivity to dust deposition^35^,^57^. Rapid drops in the number of dominant mixotrophs (*H. papilio*, *A. flavum* and *A. wrightianum*) in Linje can be connected not only with water table lowering, but also with dust deposition during local fires (see Figs 3 and 4 in Marcisz *et al*.^13^). Both factors may have led to a limiting of resources for mixotrophs which, consequently, were eliminated from the community. Moreover, mixotrophs are also influenced by light intensity, and light limitation causes a drop in their abundance^39^,^58^. The shade can be of different origin, it can be connected with air circulation, atmospheric dust deposition, more intensive growth of dwarf shrubs (such as *Ledum palustre*) in dry periods and/or local afforestation^59^. Secondary forest succession usually occurs along with water table decreases in peatlands. As it was lately shown, mixotrophs play a crucial role in C fixation and their functioning may be important for peatlands^60^. Therefore more studies on mixotrophs are needed, principally to separate the effect of light from the influence of drought.

Plagiostomic apertures were clearly connected with disturbances and the post-disturbance period (Fig. 1). Small species possessing plagiostomic apertures (*Trinema* sp. and *Corythion* sp.) were present in very low numbers during the short drought episode cal. AD 700 in Linje and were not present in PK before disturbances. They became more abundant when disturbances occurred and were established in TA communities post-disturbance. Plagiostomic apertures, small and hidden in the shell side^56^, are an adaptation trait for surviving tough conditions, e.g. periods of rapid water losses^61^, very low water tables, deforestation and mineral matter input^57^ or increased dust deposition on the peatland surface^13^,^35^. This is in line with the findings of Lamentowicz *et al*.^33^, who showed a link between the aperture type and TA adaptation to hydrological conditions. Similarly, in an experimental study in the tropical montane rainforest, Krashevska *et al*.^58^ observed that TA species with less protected terminally located apertures were significantly affected by lower water content.

## Conclusions

Under global change conditions, ecosystems are exposed to drastic shifts; hence, recognition of useful indicators of disturbances might be essential to assess the state of ecosystem functioning. We revealed that testate amoebae morphological traits were linked to environmental conditions over a long temporal perspective: (1) testate amoebae preferring dry conditions tended to prefer lower pH, possessed small shells and hidden plagiostomic apertures which are an adaptation to low water content, whereas (2) testate amoebae preferring wet conditions tended to prefer higher pH and possessed larger shells with acrostomic aperture. We underline the sensitivity of mixotrophs to abrupt disturbances. We show that species possessing plagiostomic apertures were indicators of disturbances and that their appearance during dry phases allowed their constant presence in post-disturbance communities. Moreover, disturbances caused substantial body size decreases. The above-mentioned changes in TA communities show that human impact and fires had long-term effects on peatland communities. Our results suggest a low ecosystem resilience of peatlands that – once disturbed – may not be self-restored rapidly within a few decades. Changes in trait composition affected by fire and/or peat extraction are seen in post-disturbance communities, i.e. through the establishment of new traits (plagiostomic aperture) or a lack of previously abundant traits (mixotrophy).

Our results show that testate amoebae functional traits may serve as a valuable proxy that supports conventional reconstructions of the past environmental changes in peatlands, and further analysis of trait-ecosystem relationships could make them valuable indicators of the contemporary ecosystem state. Moreover, the trait approach for testate amoebae requires more attention and more studies focused on the complex evolutionary links between testate amoebae morphology and ecosystem function.

## Additional Information

**How to cite this article**: Marcisz, K. *et al*. A novel testate amoebae trait-based approach to infer environmental disturbance in *Sphagnum* peatlands. *Sci. Rep*. **6**, 33907; doi: 10.1038/srep33907 (2016).

## Supplementary Material

Supplementary Information

## Figures and Tables

**Table 1:** Selected functional traits of testate amoebae and related detailed assumptions on how they might respond to local disturbances (drought caused by fire or peat extraction).

Trait	Description	Function(s) linked to the trait	Foreseen effect of disturbances on traits	References
Mixotrophy	Indicates presence or absence of endosymbiotic algae - whether a species is mixotroph or heterotroph (categorical variable, coded: 0-heterotroph, 1-mixotroph)	Mixotrophy is strongly related to *Sphagnum* photosynthesis, and thus C assimilation in peatlands. It can also be used as a proxy for species survival in extremely oligotrophic conditions in *Sphagnum* capitulum.	Mixotrophs require oligotrophy, high wetness and light connected with habitat openness. Dust deposition on the peatland surface (an effect of fire activity) and water table lowering harms phototrophic metabolism and leads to a decrease in the abundance of mixotrophs.	Marcisz *et al*.^13^,^39^; Krashevska *et al*.^58^; Jassey *et al*.^60^
Body size	Length of species in μm (continuous trait)	Size features of testate amoebae are linked to dissolved organic carbon and nitrogen in peatlands, and thus related to C and nitrogen cycles. Small species better overcome difficult conditions and colonize new habitats faster than large ones as they are lighter.	Shell size is correlated to moisture, therefore drought promotes higher abundance of small species.	Lamentowicz *et al*.^33^; Fournier *et al*^34^; Fiałkiewicz-Kozieł *et al*.^35^; Laggoun-Defarge *et al*.^53^; Wanner and Xylander^55^
Diameter of shell aperture	Aperture size in μm (continuous trait)		Aperture size is positively related to water availability and is linked to the feeding habits. Disturbances that cause drying promote higher abundance of species possessing small apertures.	
Body range	(body length species x – maximum body length within the community)/minimum body length within the community (calculations derived from Lamentowicz *et al*.^35^)		Small species are more resistant to rough environmental conditions. Drought promotes higher abundances of small species.	
Biovolume	Volume of a species in μm^3^ (continuous trait, calculations derived from Lamentowicz *et al*.^35^)		Shell biovolume is positively related to moisture. Drought promotes higher abundances of species with lower biovolume.	
Position of the shell aperture	(semi-continuous variable, coded: 0-axial, 1-acrostomic, 2-plagiostomic)	Unknown	Shell aperture position reflects adaptation to water availability, therefore drought or dust deposition promotes higher abundance of species possessing hidden and protected apertures.	Lamentowicz *et al*.^33^; Fiałkiewicz-Kozieł *et al*.^35^; Krashevska *et al*.^58^; Chardez and Lambert^61^

**Table 2:** Summary of the redundancy analysis results based on the testate amoeba functional trait data, macroscopic charcoal data and reconstructed water table depth.

	Linje		Puścizna Krauszowska	
	Pr (>F)	explains [%]	Pr (>F)	explains [%]
model	0.001*	41.0	0.001*	57.2
RDA axis 1	0.001*	40.5	0.001*	55.3
RDA axis 2	0.274	0.5	0.255	1.9
DWT	0.001*	35.2	0.001*	51.4
macroscopic charcoal influx	0.001*	11.5	—	—
ruderals (pollen human indicators)	—	—	0.079	3.0

Values indicate percentages of the total variance explained (adjusted R2) by the model, each ordination axis and each variable (individual fractions), and its significance (^*^Significant values, *P* < 0.01).
