# Supplementary material for: A novel testate amoebae trait-based approach to infer environmental disturbance in Sphagnum peatlands
Source: Sci Rep. 2016 Sep 23;6:33907. doi: 10.1038/srep33907 (PMC5034269; doi:10.1038/srep33907)
Supplement: Supplementary Information [file srep33907-s1.pdf]

A novel testate amoebae trait-based approach to infer environmental disturbance in *Sphagnum* peatlands

Katarzyna Marcisz, Daniele Colombaroli, Vincent E. J. Jassey, Willy Tinner, Piotr Kołaczek, Mariusz Gałka, Monika Karpińska-Kołaczek, Michał Słowiński, Mariusz Lamentowicz

Supplementary Information (including additional references)

Supplementary Methods S1. Description of peat samples preparation and identification of testate amoebae, pollen, plant macrofossils, and microscopic and macroscopic charcoal.

Testate amoebae were extracted from the peat samples following the standard protocol[1], and identified under the light microscope at 200× and 400× magnification with the help of identification keys and taxonomic monographs[2-4]. Individuals were counted until the sum of 150 was reached in each sample. Quantitative testate amoeba based depth to water table (DWT) reconstructions were calculated in C2 software[5], with the use of the training set developed for N Poland[6-8].

For the plant macrofossil analysis, sub-samples of 4cm^3 were wet sieved through a 0.2-mm mesh and identified under a binocular microscope at 200× magnification, using several macrofossil taxonomic keys[9-11].

Samples for pollen and microscopic charcoal analysis were prepared following standard preparation procedures[12]. One *Lycopodium* tablet (20,848 spores, produced by the University of Lund) was added to every sample for further calculations of pollen and microscopic charcoal concentrations ($CHAC_{micro}$)[13]. Slides were mounted in glycerine and analyzed using a light microscope at 400× and 1000× magnification. Pollen grains were counted until the sum at least 500 arboreal pollen (AP) grains was reached. The pollen taxa were identified with the assistance of available keys and atlases[14,15]. Microscopic charcoal particles > 10 μm were counted using a light microscope at 200× and 400× magnification until the sum of 200 was reached[16,17].

For macroscopic charcoal analysis, 1 cm^3 sediment samples were prepared following standard method[18]. Particles > 100 μm were counted under stereoscope at 40× magnification. Macroscopic and macroscopic charcoal influx or accumulation rates ($CHAR_{macro}$, $CHAR_{micro}$, particles/cm^2/yr) were calculated using

the charcoal concentrations ($CHAC_{macro}$, $CHAC_{macro}$), and the peat accumulation rate was inferred from the radionuclide chronology.

Supplementary Table S2. Results of ^{14}C and ^{210}Pb dating of peat sediments from Linje (S2 A, S2 B)[19] and Puścizna Krauszowska (S2 C)[20] mires. Dates were calibrated using OxCal v.4.2 program[21,22].

S2 A - Linje mire – ^{14}C dating				
Depth [cm]; in brackets a mid-point used for the age-depth model	Laboratory code – number	^{14}C date [^{14}C BP]	Callibrated age 2 σ [cal. BC/AD] Values below zero are BC dates, in brackets percentage ranges within calibrated date	Material; remarks
9–10 (9.5)	Poz-54927	106.98±0.35 pMC	1956–1957 (2.5%)<br>2002–2007 (92.9%)	*Sphagnum* stems (sect. Cuspidata); within the range of ^{210}Pb dating, excluded from the age-depth model
19–20 (19.5)	Poz-54921	120.97±0.42 pMC	1959–1961 (33%)<br>1983–1986 (62.3%)	*Sphagnum* stems (sect. Cuspidata); within the range of ^{210}Pb dating, excluded from the age-depth model
29–30 (29.5)	Poz-54926	170.2±0.44 pMC	1965–1966	*Sphagnum* stems (sect. Cuspidata); within the range of ^{210}Pb dating, excluded from the age-depth model
39–40 (39.5)	Poz-54925	113.26±0.34 pMC	1957–1958 (5.2%)<br>1991–1995 (90.2%)	*Sphagnum* stems (sect. Cuspidata); within the range of ^{210}Pb dating, excluded from the age-depth model
49–50 (49.5)	Poz-54924	175±30	1656–1697 (18%)<br>1725–1814 (51.2%)<br>1835–1878 (6%)<br>1916–>1950 (20.1%)	*Sphagnum* stems (sect. Cuspidata); within the range of ^{210}Pb dating, excluded from the age-depth model
59–60 (59.5)	Poz-54923	100±30	1682–1736 (27.1%)<br>1805–1935 (68.3%)	*Sphagnum* stems (sect. Sphagnum),
69–70 (69.5)	Poz-57452	80±30	1690–1730 (24.9%)<br>1810–1926 (70.5%)	*Sphagnum* stems
69–70 (69.5)	Poz-54919	100±30	1682–1736 (27.1%)<br>1805–1935 (68.3%)	*Sphagnum* stems
79–80 (79.5)	Poz-57453	320±25	1487–1644	*Sphagnum* stems
99–100 (99.5)	Poz-54920	790±35	1182–1280	*Sphagnum* stems, *Pinus sylvestris* - bud scale
106–107 (106.5)	Poz-54918	995±35	982–1058 (58.7%)<br>1068–1154 (36.7%)	*Sphagnum* stems,
120–122.5 (121.25)	Poz-56401	835 ±35	1057–1076 (2.6%)<br>1153–1268 (92.8%)	*Sphagnum* stems (sect. Sphagnum); outlier excluded from the age-depth model
130–132.5 (131.25)	Poz-56402	1145±35	776–978	*Sphagnum* stems (sect. Sphagnum)
140–142.5 (141.25)	Poz-56403	1345±30	640–714 (86.4%)<br>744–765 (9%)	*Sphagnum* stems (sect. Sphagnum)
150–152.5 (151.25)	Poz-56405	1500±30	432–489 (10.6%)<br>532–638 (84.8%)	*Sphagnum* stems (sect. Sphagnum)
160–162.5 (161.25)	Poz-56406	1525±30	428–498 (35.1%)<br>504–604 (60.3%)	*Sphagnum* stems (sect. Sphagnum)
170–172.5 (171.25)	Poz-56407	1535±35	426–598	*Sphagnum* stems (sect. Sphagnum)
180–182.5 (181.25)	Poz-56408	1510±35	428–494 (23.2%)<br>507–520 (2.4%)<br>526–634 (69.8%)	*Sphagnum* stems (sect. Sphagnum)
190–192.5 (191.25)	Poz-56409	1635±30	427–592	*Sphagnum* stems (sect. Sphagnum)
200–202.5 (201.25)	Poz-56410	1945±30	-21–-11 (1.8%)<br>-2–128 (93.5%)	*Sphagnum* stems (sect. Sphagnum)
210–2012.5 (211.25)	Poz-56411	2025±30	-151–-143 (0.9%)<br>-112–-55 (94.5%)	*Sphagnum* stems (sect. Sphagnum)

S2 B - Linje mire - ^{210}Pb dating	
Depth for layer middle (cm)	Age for layer middle (y)
2	2011 ± 0.2
4.5	2009 ± 0.2
5.5	2007 ± 0.5
6.5	2006 ± 0.5
7.5	2004 ± 0.5
8.5	2003 ± 0.5
9.5	2002 ± 0.5
10.5	2001 ± 0.8
11.5	1999 ± 0.8
12.5	1996 ± 0.8
13.5	1994 ± 0.9
14.5	1992 ± 0.9
15.5	1991 ± 0.9
16.5	1989 ± 1.2
17.5	1986 ± 1.2
18.5	1985 ± 1.5
19.5	1983 ± 1.5
20.5	1982 ± 1.6
21.5	1980 ±1.6
22.5	1978 ± 1.6
23.5	1977 ± 1.6
24.5	1976 ± 1.8
25.5	1974 ± 2
26.5	1971 ± 2
27.5	1969 ± 2
28.5	1967 ± 2
29.5	1966 ± 2
30.5	1964 ± 2
31.5	1962 ± 2
32.5	1958 ± 3
33.5	1956 ± 3
34.5	1953 ± 3
35.5	1951 ± 3
36.5	1948 ± 4
37.5	1946 ± 4
38.5	1943 ± 4
39.5	1940 ± 5
40.5	1938 ± 5
41.5	1936 ± 6
42.5	1934 ± 6
43.5	1931 ± 7
44.5	1929 ± 7
45.5	1926 ± 8
46.5	1922 ± 10
47.5	1918 ± 10
48.5	1913 ± 11
49.5	1909 ± 12
50.5	1905 ± 14
51.5	1901 ± 16
52.5	1895 ± 19
53.5	1886 ± 25
54.5	1878 ± 31
55.5	1869 ± 38
56.5	1856 ± 59
57.5	1835 ± 89

S2 C - Puścizna Krauszowska mire – ^{14}C dating					
Depth [cm]	Laboratory code – number	Uncalibrated age [yr ^{14}C BP]	Calibrated [cal. BC/AD] (Values unrounded)	Modelled age (μ ± σ or 68.2% interval) [cal. AD/-BC]	Remarks
18.5	GdS-973	146 ± 4.1 pMC	68.2% interval:<br>1962e1963 (3.4%)<br>1971e1974 (64.8%)<br>95.4% interval:<br>1962e1963 (10.6%)<br>1969e1975 (84.8%)	1970 ± 5	outlier excluded from the age-depth model
24.5	GdS-897	1355 ± 40	68.2% intervals:<br>640e689 (64.8%)<br>752e759 (3.4%)<br>95.4% intervals:<br>610e725 (85.1%)<br>741e767 (10.3%)	-	outlier excluded from the age-depth model
30.5	GdS-933	949 ± 57	68.2% interval:<br>1026e1059 (18.9%)<br>1065e1154 (49.7%)<br>95.4% interval:<br>994e1209 (95.4%)	-	outlier excluded from the age-depth model
36.5	GdS-941	430 ± 60	68.2% intervals:<br>1420e1514 (60.1%)<br>1600e1617 (8.1%)<br>95.4% interval:<br>1406e1531 (95.4%)<br>1539e1635 (26.9%)	-	outlier excluded from the age-depth model
42.5	GdS-942	465 ± 60	68.2% intervals:<br>1402e1488 (66.7%)<br>1604e1608 (1.5%)<br>95.4% intervals:<br>1316e1355 (6.7%)<br>1389e1524 (77.3%)<br>1558e1632 (11.4%)	-	outlier excluded from the age-depth model
48.5	GdS-934	1388 ± 52	68.2% interval:<br>604e674 (68.2%)<br>95.4% intervals:<br>560e714 (91.4%)<br>744e765 (4.0%)	630e690 (55.4%)<br>745e765 (12.8%)	
54.5	GdS-935	1361 ± 53	68.2% intervals:<br>619e690 (62.4%)<br>750e761 (5.8%)<br>95.4% interval:<br>587e770 (95.4%)	615 ± 40	
60.5	GdS-936	1634 ± 58	68.2% intervals:<br>344e434 (42.6%)<br>454e470 (5.8%)<br>487e534 (19.9%)<br>95.4% intervals:<br>256e300 (6.0%)<br>318e556 (89.4%)	400e430 (10.6%)<br>450e530 (50.5%)	
66.5	GdS-943	1780 ± 70	68.2% intervals:<br>140e197 (18.9%)<br>208e333 (49.3%)<br>95.4% interval:<br>84e395 (95.4%)	270 ± 75	
72.5	GdS-944	1590 ± 75	8.2% interval:<br>395e560 (68.2%)<br>95.4% intervals:<br>259e281 (1.9%)<br>324e621 (93.5%)	465 ± 80	outlier excluded from the age-depth model
90.5	GdS-945	2000 ± 80	8.2% interval:<br>-111e83 (68.2%)<br>95.4% interval:<br>-203e214 (95.4%)	-10 ± 75	
99.5	GdS-937	2060 ± 100	8.2% intervals:<br>-199e 52 (68.2%)<br>95.4% interval:<br>-361e126 (95.4%)	-60 ± 90	

Supplementary Figure S3. Age-depth models constructed for Linje[19] and Puścizna Krauszowska[20].

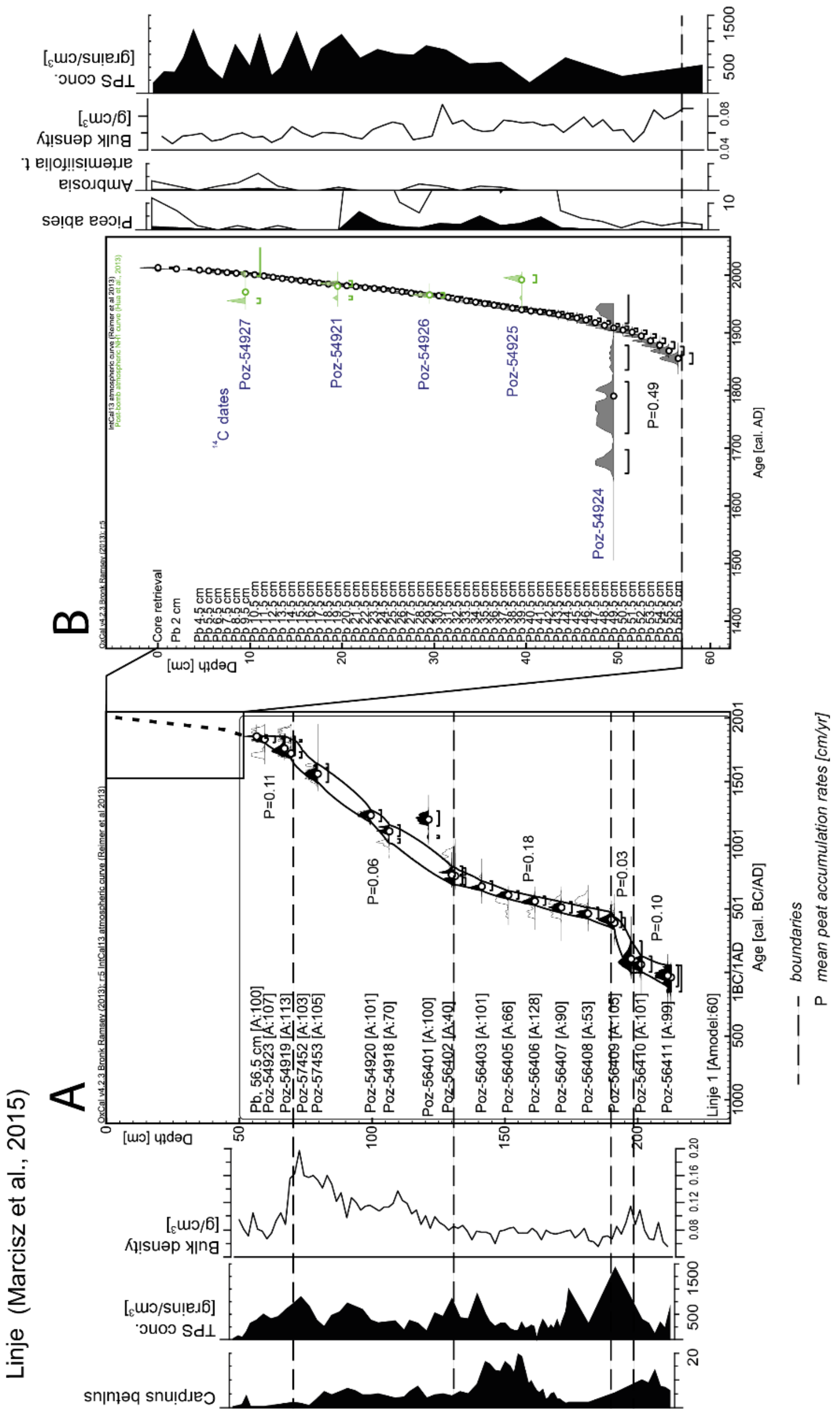

Puścizna Krauszowska (Fiałkiewicz-Kozieł et al., 2015)

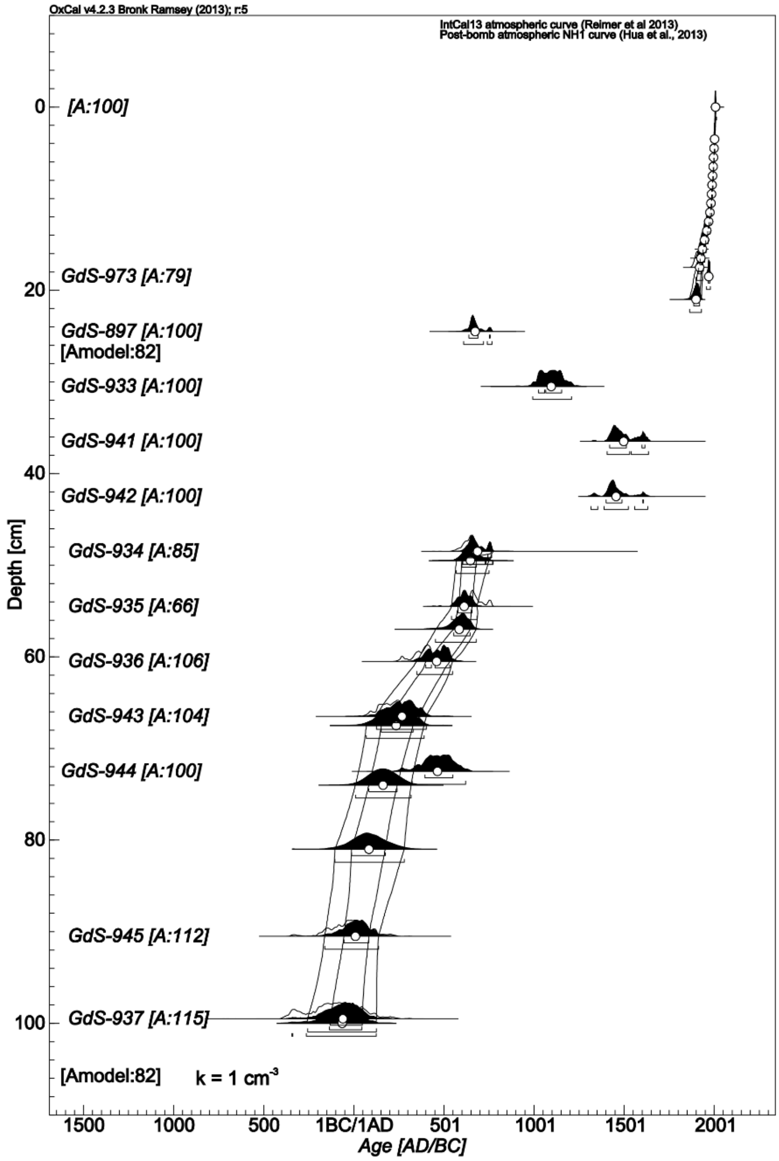

Supplementary Table S4. Selected functional groups of testate amoebae species for which the values of *diversity indices* were calculated along the sequences. Ecological preferences (preferred hydrological conditions and pH), were assessed according to the study by Lamentowicz and Mitchell [6], where these preferences were recognized for TA species from Polish peatlands.

list of testate amoeba taxa	Shape of the shell					Ecological preferences					Present in	
	Size		Aperture position			Preferred pH		Hydrological preferences			Linje mire	Puścizna Krauszowska mire
	small (< 50 µm)	large (> 50 µm)	acrostomic	plagiostomic	axial	pH < 4	pH > 4	wet	intermediate	dry		
Amphitrema wrightianum		x	x			x		x			x	
Arcella artocrea		x			x						x	
Arcella catinus		x			x		x		x		x	x
Arcella discoides		x			x		x		x		x	x
Arcella hemispherica		x			x		x	x				x
Arcella sp.		x			x						x	x
Archerella flavum		x	x				x	x			x	x
Assulina muscorum	x		x			x				x	x	x
Assulina scandinavica		x	x			x				x		x
Assulina seminulum		x	x			x				x	x	x
Bullinularia indica		x		x		x				x	x	x
Centropyxis aculeata		x		x			x	x			x	
Centropyxis aerophila		x		x			x		x		x	
Centropyxis aerophila sphagnicola		x		x							x	
Centropyxis constricta		x		x							x	
Centropyxis laevis		x		x							x	
Centropyxis orbicularis		x		x							x	
Corythion dubium	x			x		x				x	x	x
Cyclopyxis arcelloides		x			x		x	x			x	x
Cyclopyxis sp.		x			x						x	
Cryptodifflugia crenulata	x		x							x	x	
Cryptodifflugia oviformis	x		x			x				x	x	
Difflugia globulosa		x	x			x		x			x	x
Difflugia leidyi		x	x				x	x			x	
Difflugia oblonga		x	x				x	x				x
Difflugia urceollata		x	x								x	
Difflugia sp.		x	x								x	
Euglypha ciliata		x	x			x	x				x	
Euglypha compressa		x	x				x	x			x	x
Euglypha compressa glabra		x	x								x	
Euglypha cristata	x		x				x			x	x	x
Euglypha hyalina/rotunda type	x		x			x				x	x	x
Euglypha tuberculata		x	x			x				x		x
Euglypha sp.		x	x								x	x

Euglypha strigosa		x	x			x				x	x	
Heleopera petricola		x	x				x		x		x	x
Heleopera rosea		x	x				x		x		x	
Heleopera sphagni		x	x				x		x		x	x
Heleopera sylvatica		x	x			x				x		x
Hyalosphenia elegans		x	x			x		x			x	x
Hyalosphenia papilo		x	x				x	x			x	x
Hyalosphenia subflava		x	x				x		x			x
Nebela carinata		x	x				x	x			x	
Nebela marginata		x	x								x	
Nebela militaris		x	x			x			x		x	x
Nebela tincta s.l.		x	x			x				x	x	x
Nebela tincta major		x	x			x				x	x	
Nebela sp.		x	x								x	x
Phryganella acropodia	x				x		x	x			x	x
Physochila griseola		x	x			x			x		x	x
Placocista spinosa		x	x								x	
Trigonopyxis arcula		x			x	x				x	x	x
Trinema enchelys	x			x		x				x	x	x
Trinema lineare	x			x		x				x	x	x

Additional References

1 Booth, R. K., Lamentowicz, M. & Charman, D. J. Preparation and analysis of testate amoebae in peatland paleoenvironmental studies. *Mires and Peat* **7 (2010/11)**, 1-7 (2010).

2 Ogden, C. G. & Hedley, R. H. *An Atlas of Freshwater Testate Amoebae*. (Oxford University Press, 1980).

3 Meisterfeld, R. in *Patrimoines Naturels* Vol. 50 (eds M. J. Costello, C. S. Emblow, & R. White) 54-57 (Muséum National d'Histoire Naturelle - Institut d'Ecologie et de Gestion de la Biodiversité (I.E.G.B.) - Service du Patrimoine Naturel (S.P.N.), 2001).

4 Mazei, Y. & Tsyganov, A. N. *Freshwater testate amoebae*. (KMK, 2006).

5 Juggins, S. *C2 User Guide. Software for Ecological and Palaeoecological Data Analysis and Visualisation*. 69 (2003).

6 Lamentowicz, M. & Mitchell, E. A. D. The ecology of testate amoebae (Protists) in Sphagnum in north-western Poland in relation to peatland ecology. *Microbial Ecology* **50**, 48-63 (2005).

7 Lamentowicz, M. & Mitchell, E. A. D. Testate amoebae (Protists) as palaeoenvironmental indicators in peatlands. *Polish Geological Institute Special Papers* **16**, 58-64 (2005).

8 Lamentowicz, M. *et al.* Last millennium palaeoenvironmental changes from a Baltic bog (Poland) inferred from stable isotopes, pollen, plant macrofossils and testate amoebae. *Palaeogeography, Palaeoclimatology, Palaeoecology* **265**, 93-106 (2008).

9 Tobolski, K. *Vademecum Geobotanicum*. (Wydawnictwo Naukowe PWN, 2000).

10 Laine, J. & laitos, H. Y. M. *The Intricate Beauty of Sphagnum Mosses: A Finnish Guide for Identification*. (Department of Forest Ecology, University of Helsinki, 2009).

11 Grosse-Brauckmann, G. Über pflanzliche Makrofossilien mitteleuropäischer Torfe. III. Früchte, Samen und einige Gewebe (Fotos von fossilen Pflanzenresten) - On plant macrofossils in central European peat. III. Fruits, seeds and some tissues (photos of fossil plant remains) (in German). *Telma* **22**, 53-102 (1992).

12 Berglund, B. E. & Ralska-Jasiewiczowa, M. in *Handbook of Holocene Palaeoecology and Palaeohydrology* (ed B. E. Berglund) 455-484 (John Wiley & Sons, Chichester, 1986).

13 Stockmarr, J. Tablets with spores used in absolute pollen analysis. *Pollen et Spores* **13**, 615-621 (1971).

14 Beug, H.-J. *Leitfaden der Pollenbestimmung für Mitteleuropa und angrenzende Gebiete*. (Verlag Dr. Friedrich Pfeil, 2004).

15 Moore, P. D., Webb, J. A. & Collinson, M. E. *Pollen Analysis*. (Blackwell Scientific Publications, Oxford, 1991).

16 Tinner, W. & Hu, F. S. Size parameters, size-class distribution and area-number relationship of microscopic charcoal: relevance for fire reconstruction. *The Holocene* **13**, 499-505 (2003).

17 Finsinger, W. & Tinner, W. Minimum count sums for charcoal-concentration estimates in pollen slides: accuracy and potential errors. *The Holocene* **15**, 293-297 (2005).

18 Whitlock, C. & Larsen, C. in *Tracking environmental change using lake sediments. Terrestrial, algal, and siliceous indicators. J. P. Smol, H. J. B. Birks, and W. M. Last, Eds.* Vol. 3 75-97 (2001).

19 Marcisz, K. *et al.* Long-term hydrological dynamics and fire history over the last 2000 years in CE Europe reconstructed from a high-resolution peat archive. *Quaternary Science Reviews* **112**, 138-152, doi:10.1016/j.quascirev.2015.01.019 (2015).

20 Fiałkiewicz-Kozieł, B., Kołaczek, P., Michczyński, A. & Piotrowska, N. The construction of a reliable absolute chronology for the last two millennia in an anthropogenically disturbed peat bog: Limitations and advantages of using a radio-isotopic proxy and age-depth modelling. *Quaternary Geochronology* **25**, 83-95 (2015).

21 Bronk Ramsey, C. Radiocarbon calibration and analysis of stratigraphy: The OxCal program. *Radiocarbon* **37**, 425-430 (1995).

22 Bronk Ramsey, C. Deposition models for chronological records. *Quaternary Science Reviews* **27**, 42-60 (2008).
